# Recurrent respiratory papillomatosis juvenile: a retrospective analysis at Hospital Santo Antônio

**DOI:** 10.1016/j.bjorl.2026.101943

**Published:** 2026-10-31

**Authors:** Poliana Brito Barbosa, Dário Oliveira Lopes, Alana Mendonça Amorim, Gustavo Barreto Cunha, Tiago Silva Almeida, Fátima Matos

**Affiliations:** a Universidade Federal da Bahia (UFBA), Salvador, BA, Brazil; b Obras Sociais Irmã Dulce (OSID), Salvador, BA, Brazil; c Santa Casa de Misericórdia da Bahia (SCMBA), Salvador, BA, Brazil; d Escola Bahiana de Medicina e Saúde Pública (EBMSP), Salvador, BA, Brazil; e Universidade Federal de Pernambuco (UFPE), Recife, PE, Brazil

**Keywords:** Juvenile recurrent respiratory papillomatosis, Human papillomavirus, Laryngeal papillomatosis, Derkay score, HPV

## Abstract

**Objective:**

To describe the profile of patients with Recurrent respiratory papillomatosis juvenile treated at a Brazilian tertiary referral center, including disease severity assessed with the Derkay staging system.

**Methods:**

This retrospective observational case series included patients younger than 18 years with histopathologically confirmed who underwent laryngeal surgery and outpatient follow-up. Medical records from January 2014 to December 2025 were reviewed. Demographic, clinical, surgical, recurrence, airway, adjuvant-treatment, mortality, and Derkay-score data were analyzed descriptively.

**Results:**

Seventeen patients were included; 10 were female (58.8%) and 7 were male (41.2%). The mean age at symptom onset was 5.23 ± 3.73 years, and isolated dysphonia was the most common initial presentation (70.6%). Dysphonia associated with dyspnea occurred in 29.4% of patients. A total of 53 surgical procedures were performed, corresponding to a mean of 3.12 procedures per patient. Disease recurrence occurred in 12 patients (70.6%), and 4 patients (23.5%) required tracheostomy. One patient with extensive tracheal involvement received intralesional and systemic bevacizumab, with subsequent decannulation and no need for repeat tracheostomy. Two patients died from disease-related complications. Among 12 patients with serial Derkay assessments, the score decreased in 11 (91.7%) and remained stable in 1 (8.3%); the mean total score declined from 10.94 to 7.00 points.

**Conclusions:**

Recurrent respiratory papillomatosis juvenile was associated with substantial morbidity, frequent recurrence, repeated surgical interventions, tracheostomy, and mortality in this Brazilian pediatric cohort. Dysphonia should prompt early specialized evaluation, particularly when accompanied by dyspnea. Limited access to CO_2_ laser, pediatric bronchoscopy, HPV subtyping, and adjuvant therapies may increase the clinical burden in public referral settings. Multicenter studies are needed to clarify prognostic factors and evaluate the impact of HPV vaccination and adjuvant treatments. National data from Brazilian public referral centers remain limited.

**Level of evidence:**

4.

## Highlights

• Retrospective series of 17 children with recurrent respiratory papillomatosis.
• Recurrence occurred in 71% of patients despite multiple surgical interventions.
• Tracheostomy was required in 24% of cases, reflecting high disease severity.
• Public centers face limited access to laser, subtyping, and adjuvant therapies.
• Findings support HPV vaccination as adjuvant therapy in recurrent cases.

## Introduction

Recurrent Respiratory Papillomatosis (RRP) is a rare benign disease of the airways characterized by the formation of exophytic epithelial lesions, associated primarily with Human Papillomavirus (HPV), particularly subtypes 6 and 11. Although histologically benign, the disease exhibits potentially aggressive clinical behavior due to a high recurrence rate and the possibility of progressive airway obstruction.^1^ RRP is commonly classified based on age at onset and disease severity. With respect to age at onset, it can be classified as juvenile or adult. The juvenile form (juvenile recurrent respiratory papillomatosis) generally manifests before 18-years of age and tends to present greater clinical aggressiveness, frequently requiring multiple surgical interventions throughout childhood. The adult form, in turn, typically has a less aggressive course and a lower frequency of recurrences. The adult form is defined by disease onset after 18-years.^2^

Regarding etiology, clinical experiments and studies using light and electron microscopy have suggested an association of recurrent laryngeal papillomatosis with viral infections. Only with the development of molecular biology and different techniques of viral DNA hybridization was it possible to demonstrate this relationship, which was confirmed by the PCR (polymerase chain reaction) technique. The main cause is infection by HPV, especially types 6 and 11. The disease is characterized by proliferative lesions that may recur. The main treatment of recurrent laryngeal papillomatosis is surgical, with removal of the papillomas.

Despite therapeutic advances, management of the disease still represents a significant clinical challenge, especially in public health systems with limited access to technologies such as CO_2_ lasers, microdebrider, specialized pediatric bronchoscopy, viral typing methods and and adjuvant therapies, such as the use of bevacizumab.

International studies have investigated the potential impact of vaccination on reducing disease incidence, although demonstrating a causal association still requires robust epidemiological studies. In Brazil, the quadrivalent HPV vaccine was introduced in 2014 in the National Immunization Schedule.^3^ It protects against four viral subtypes: 6, 11, 16 and 18. Subtypes 6 and 11 are mainly associated with the development of genital warts and recurrent laryngeal papillomatosis.

Recently, the group of patients with recurrent laryngeal papillomatosis was designated as a priority group to receive the HPV vaccine,^4^ following publications that demonstrated the benefits of the vaccine as an adjuvant treatment for these patients,^5^ indicating a significant reduction in the number and in the interval of recurrences in immunized patients.^6^^,^^7^

Hospital Santo Antônio is a tertiary referral center for head and neck diseases in the state of Bahia, receiving pediatric patients with recurrent respiratory papillomatosis from different regions of the state.

Given the high morbidity associated with the disease and the scarcity of recent national data on the experience of Brazilian public centers, this study aimed to describe the clinical, therapeutic, and evolutionary profile of pediatric patients with juvenile recurrent respiratory papillomatosis followed at a Brazilian tertiary center between 2014 and 2025.

## Methods

### Study design

This was a retrospective, observational, case series study conducted in the Department of Head and Neck Surgery of Hospital Santo Antônio.

### Ethical considerations

The study was approved by the institution's Research Ethics Committee, in accordance with the ethical principles of the Declaration of Helsinki and national regulations for research involving human subjects. Data were analyzed anonymously, in compliance with the General Data Protection Law (LGPD).

### Study population

We reviewed the medical records of patients who underwent laryngeal surgical procedures between January 2014 and December 2025, identified using SIGTAP codes (it is a procedure coding system of the Brazilian SUS) related to microlaryngeal procedures: SIGTAP: 04.04.01.011-3 – EXCISION OF LARYNGEAL PAPILLOMA, 04.04.01.017-2 – PARTIAL LARYNGECTOMY, 04.16.03.025-4 – PARTIAL LARYNGECTOMY IN ONCOLOGY. The SIGTAP codes initially used searched the database for all patients who had undergone partial laryngectomy surgeries, since it was not possible to filter by date of birth or type of procedure due to technical limitations. This larger initial set was intended to ensure that no pediatric patient was excluded from the study.

Initially, 325 medical records were identified. After individual review of the records, 17 pediatric patients with presumed diagnosis of recurrent laryngeal papillomatosis were selected. The STROBE flowchart model was used for sample selection.

The following inclusion and exclusion criteria were applied, and the following variables were analyzed: patients under 18-years of age at the time of diagnosis, with pathologically confirmed diagnosis of Recurrent Respiratory Papillomatosis (RRP), outpatient follow-up during the study period, and medical records containing the minimum clinical information necessary for analysis. Patients with onset of papillomatosis in adulthood, incomplete records, and cases without histopathological confirmation were excluded. The variables analyzed included sex, age at symptom onset, initial symptoms, number of surgical procedures, presence of recurrence, need for tracheostomy, tracheal dissemination, use of adjuvant therapies, and occurrence of death. The Derkay^8^ staging system, which quantifies the severity of Recurrent Respiratory Papillomatosis (RRP), was also used.

### Operational definitions

Recurrence was defined as the reappearance of laryngeal lesions after previously documented surgical treatment.

Cases were considered clinically aggressive when they required multiple surgical procedures, significant respiratory compromise, tracheobronchial dissemination, or the need for tracheostomy.

The indication for tracheostomy was determined on a case-by-case basis by the attending team in situations of significant airway obstruction or the need for multiple interventions in a short period of time.

### Clinical evaluation

Patients were evaluated in the outpatient clinic pre- and postoperatively using a 70° rigid laryngoscope and/or a flexible nasofibrolaryngoscope connected to a video system for lesion documentation, anatomic assessment, and clinical follow-up.

All patients underwent surgical resection of the lesions by direct laryngoscopy using conventional technique with cold instruments. The service did not have access to a CO_2_ laser during the study period, nor did it perform HPV subtyping, due to unavailability within the SUS network.

### Statistical analysis

The data were analyzed using descriptive statistics. Continuous variables were expressed as mean and standard deviation, while categorical variables were presented as absolute and relative frequencies.

## Results

### Demographic characteristics

Seventeen pediatric patients with a confirmed diagnosis of juvenile recurrent respiratory papillomatosis were included. It is important to note that no mother had been vaccinated against HPV, nor had any child at the time of diagnosis and at the time of the first procedure.

There was a predominance of the female sex, corresponding to 10 patients (58.82%), while 7 patients (41.18%) were male.

The age of symptom onset ranged from 1 to 14 years, with a mean of 5.23 ± 3.73 years, and most patients underwent their first surgery before 5-years of age.

### Clinical manifestations

Isolated dysphonia was the most common initial symptom, observed in 12 patients (70.59%). Five patients (29.41%) presented dysphonia associated with dyspnea. During initial evaluation in emergency services, some patients received preliminary diagnoses of bronchiolitis, asthma, or pneumonia before undergoing specialized endoscopic assessment. Only after more detailed examinations, due to worsening dyspnea or orthopnea, were the patients referred for fiberoptic nasolaryngoscopy.

### Surgical treatment

All patients underwent surgical resection of the lesions by direct laryngoscopy using cold instruments. During the study period, 53 surgical procedures were performed, with a mean of 3.12 surgeries per patient.

Representative cases followed during the study period are presented below (Fig. 1).

**Fig. 1:**
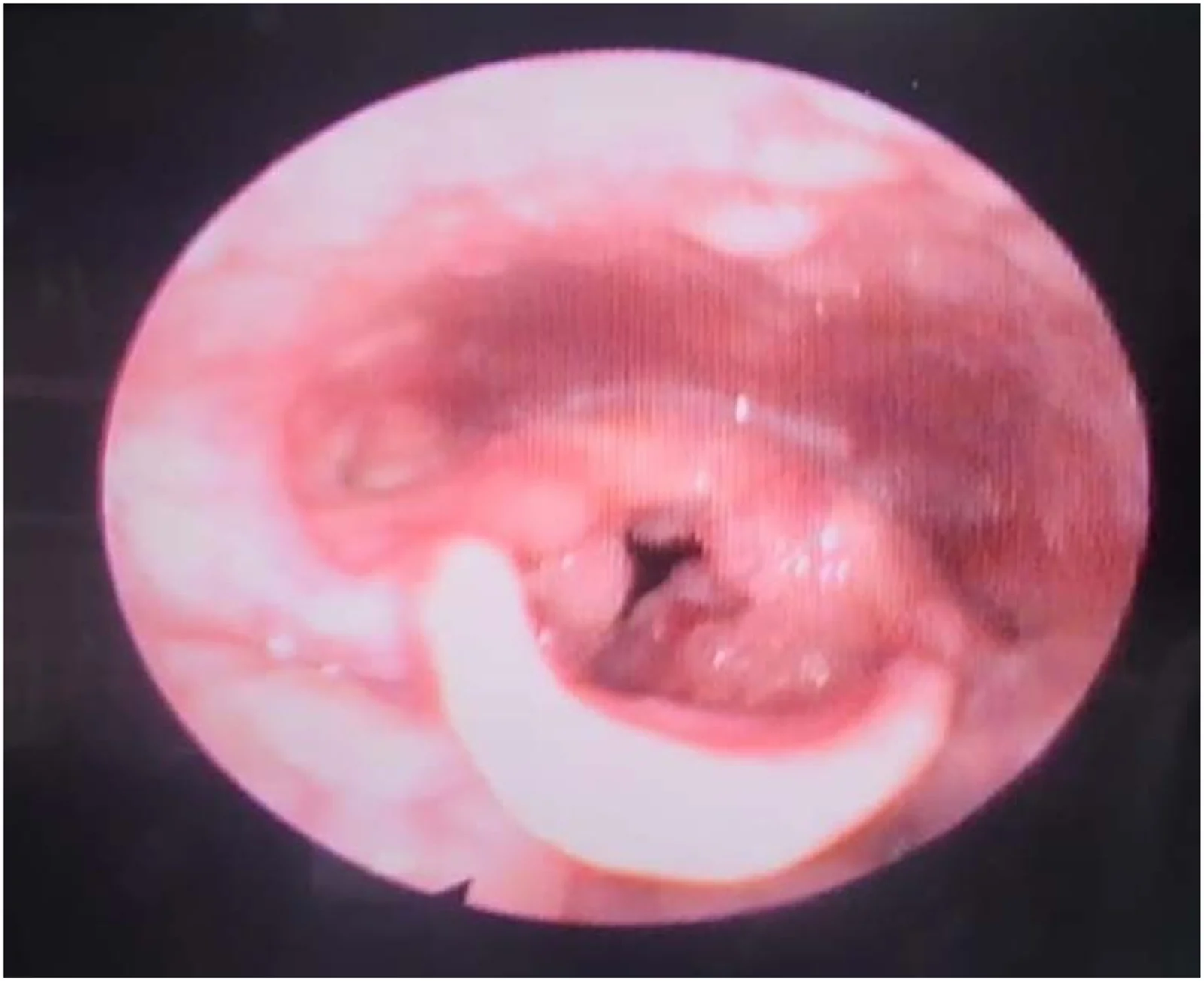
Appearance before the first surgery in 2019. Patient aged 8-months. Source: Personal collection.

The patient continued to be followed in our service, requiring multiple resections. Tracheostomy was necessary due to the need for monthly procedures, given the severity of the recurrence.

The lesions continued to recur, eventually leading to extensive involvement of the tracheal lumen and the need for referral to a service that offered pediatric bronchoscopy and subsequent treatment with adjuvant therapies (Fig. 2, Fig. 3).

**Fig. 2:**
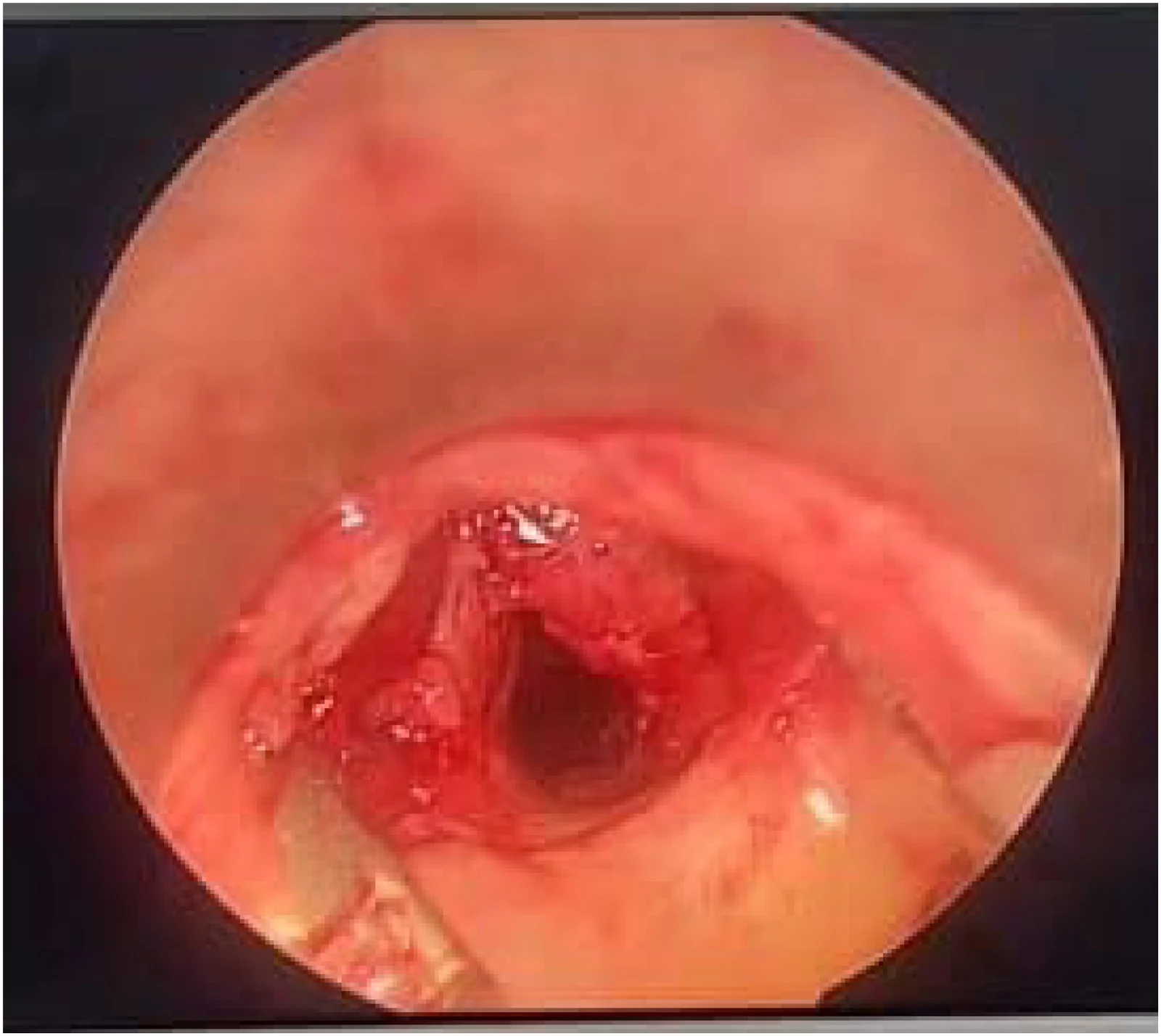
Appearance after multiple resections in 2020 and tracheal invasion. Patient aged 1.5-years. Source: Personal collection.

**Fig. 3:**
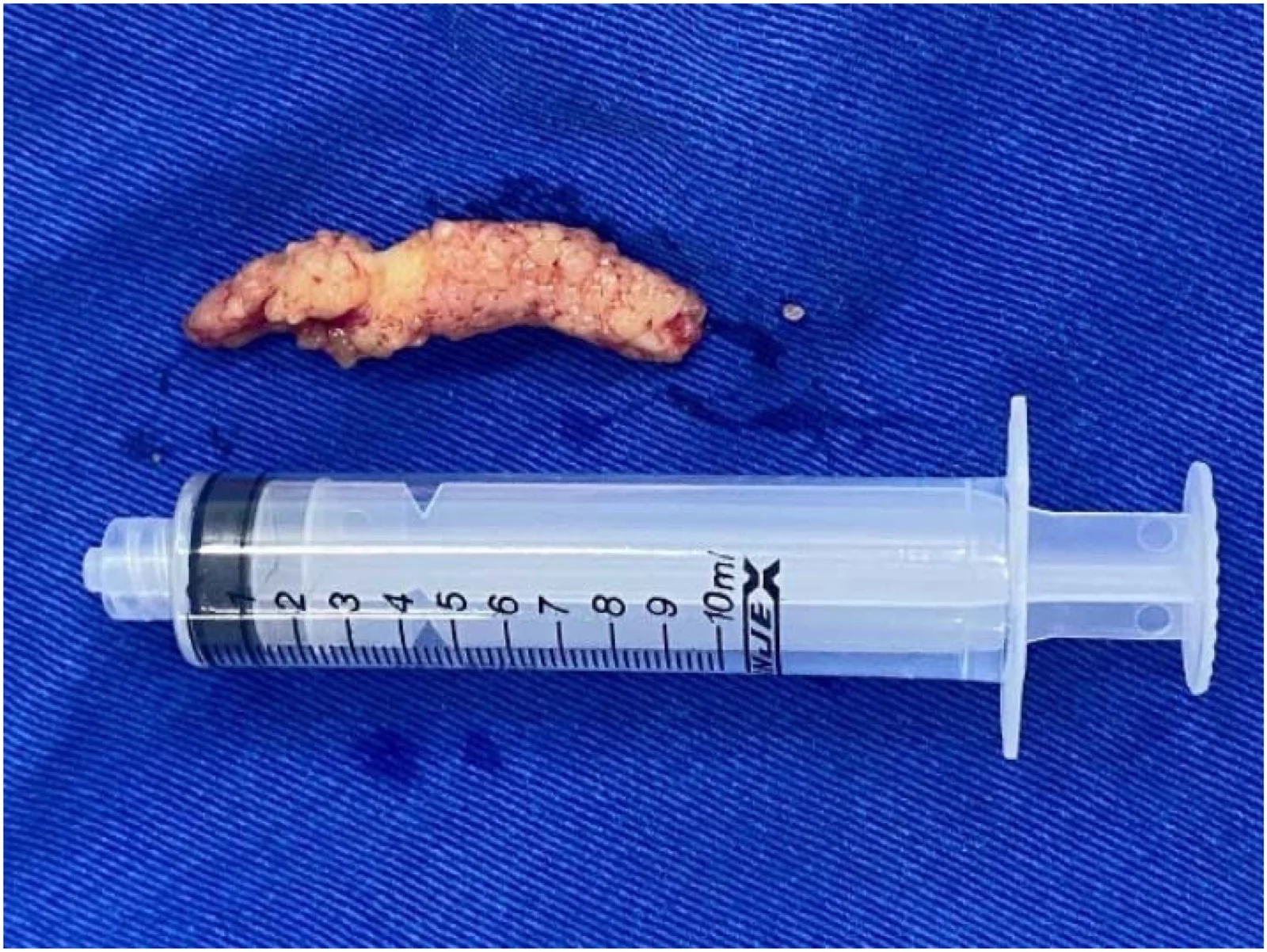
Extensive involvement of the tracheal lumen in 2020. Patient aged 18-months. Source: Personal collection.

### Recurrence

Disease recurrence was observed in 12 patients (70.59%).

Younger patients required repeated interventions at shorter intervals, especially in cases with more severe respiratory compromise.

The patient below has been followed in our clinic for 7-years and underwent a total of 8 procedures. The image below shows the laryngoscopy before the first procedure. There is significant obstruction at the glottic level due to the papillomas, with a small glottic opening (Fig. 4).

**Fig. 4:**
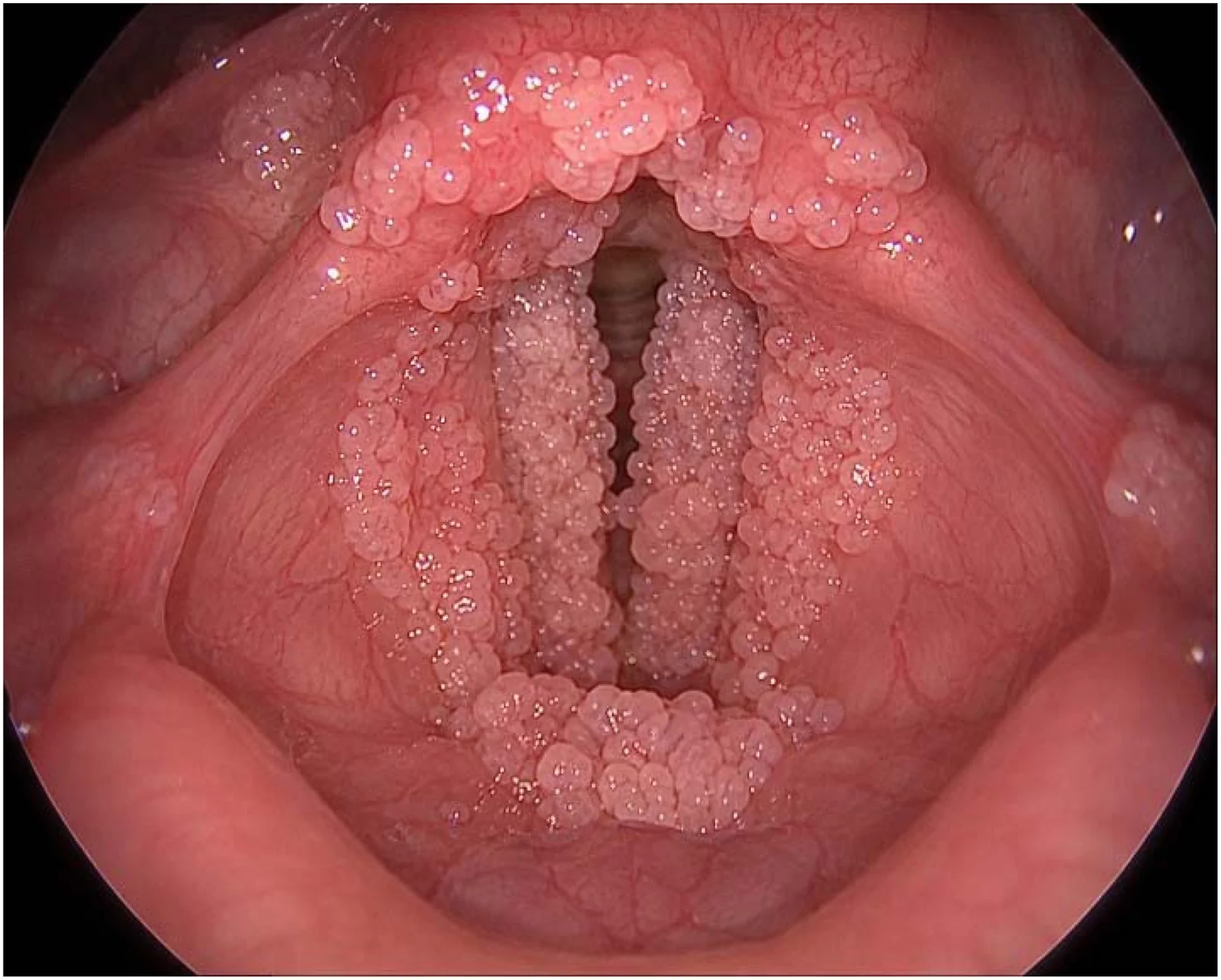
Appearance before the first procedure in 2018, patient aged 3-years. Reproduction based on the real image.

The patient continued to be followed, with the last follow-up in 2025. He underwent, once again, surgical excision of the papillomas. No adjuvant therapy was used due to unavailability at the service, but the patient would be a candidate for the use of bevacizumab (Fig. 5, Fig. 6).

**Fig. 5:**
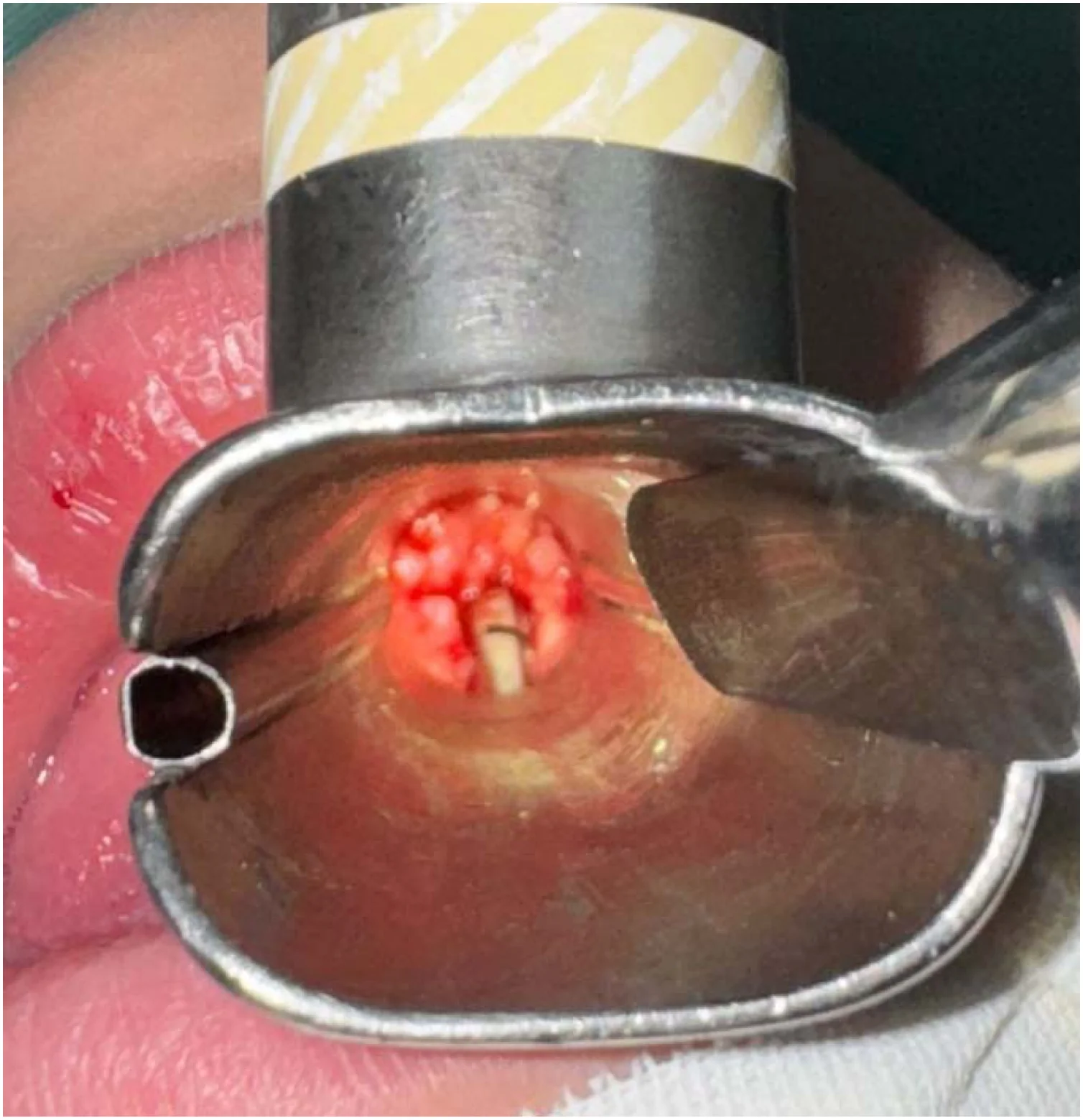
Appearance before the 8th procedure in 2025, patient aged 10-years. Source: Personal collection.

**Fig. 6:**
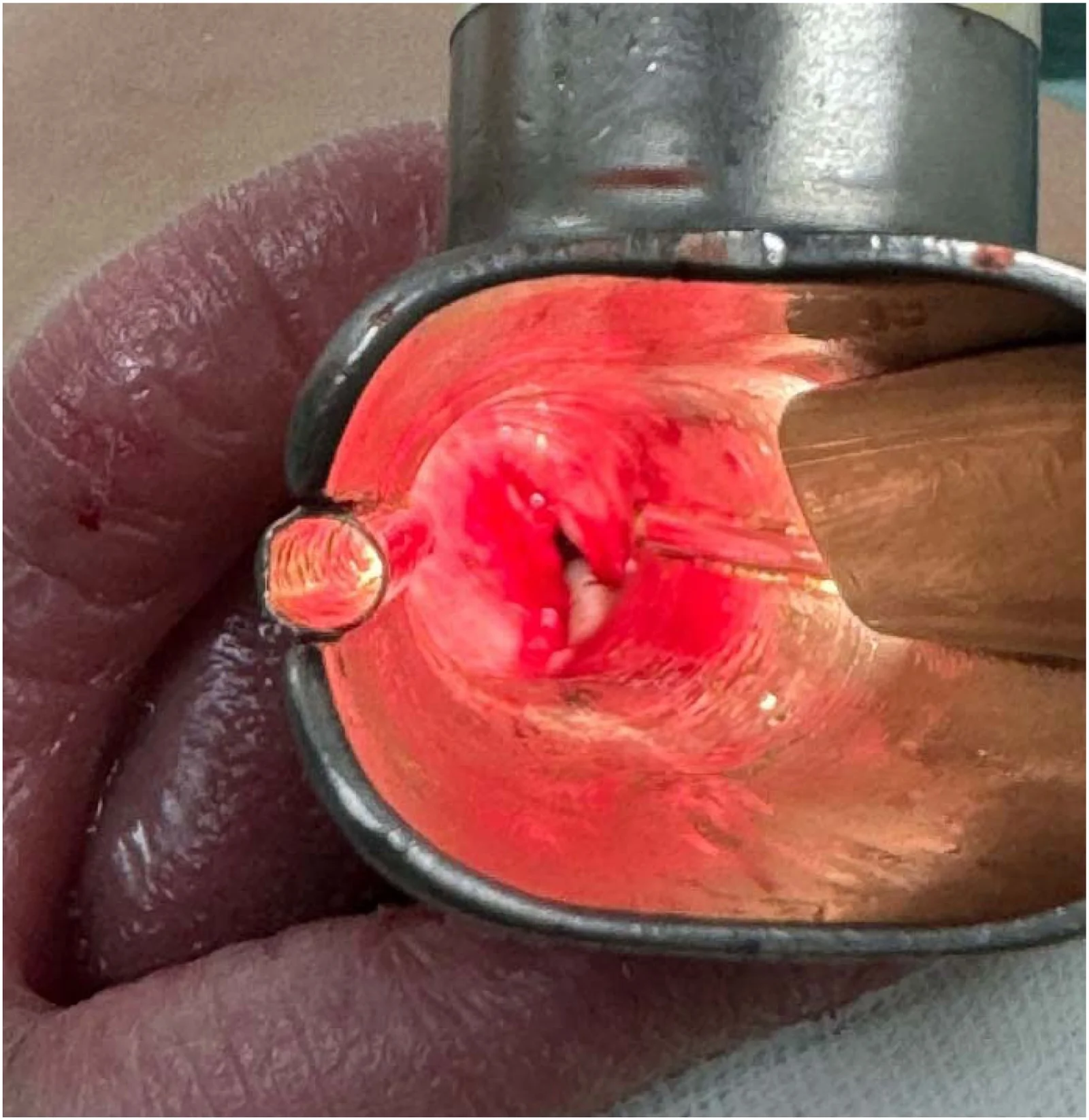
Immediate postoperative appearance after the 8th procedure in 2025. Source: Personal collection.

Despite the service not having HPV subtyping, below is the slide referring to the case, with the classic characteristics of HPV lesions, such as the presence of papillae, atypical nuclei and koilocytosis (Fig. 7, Fig. 8).

**Fig. 7:**
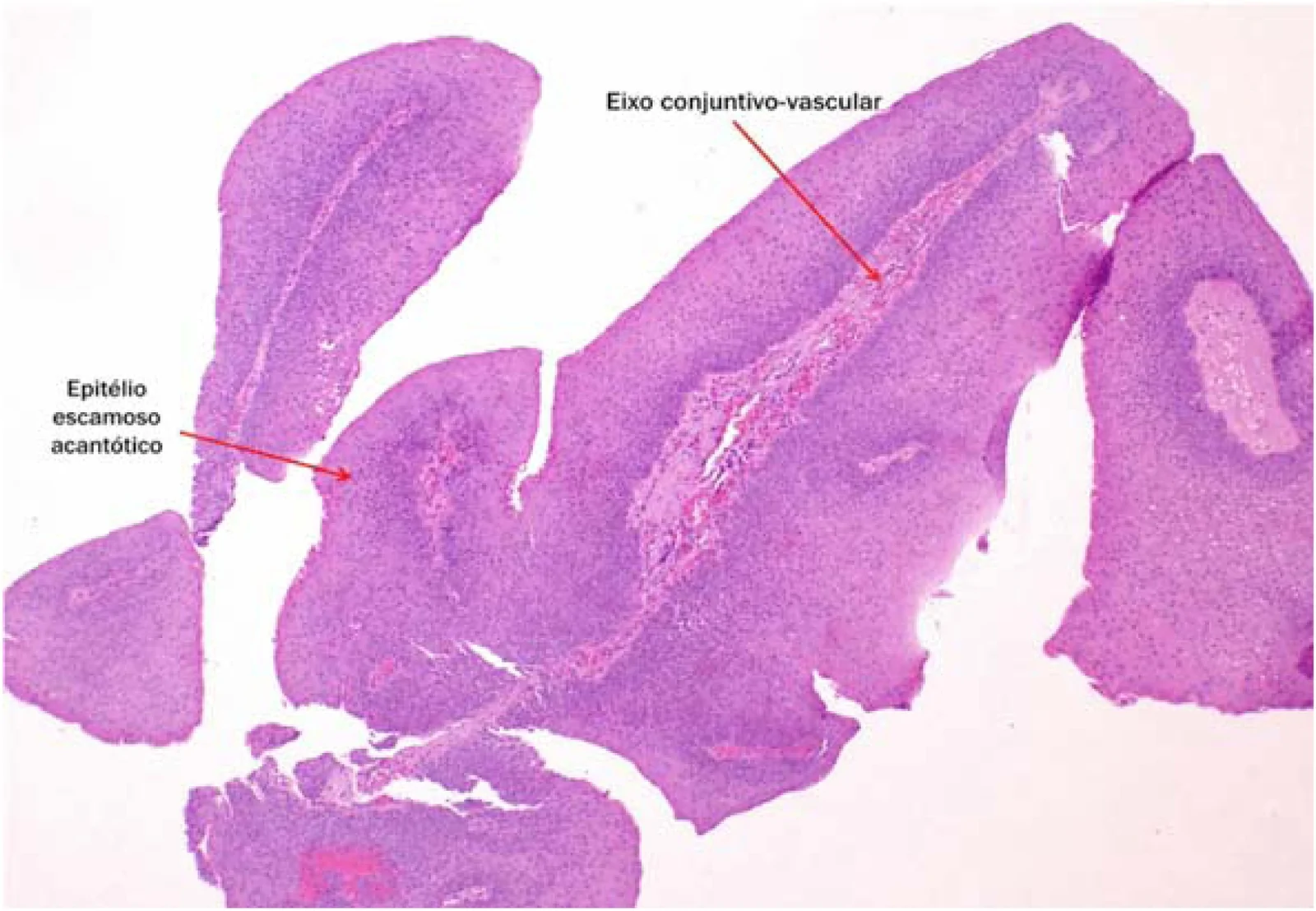
Histological image of the patient. Source: Personal collection.

**Fig. 8:**
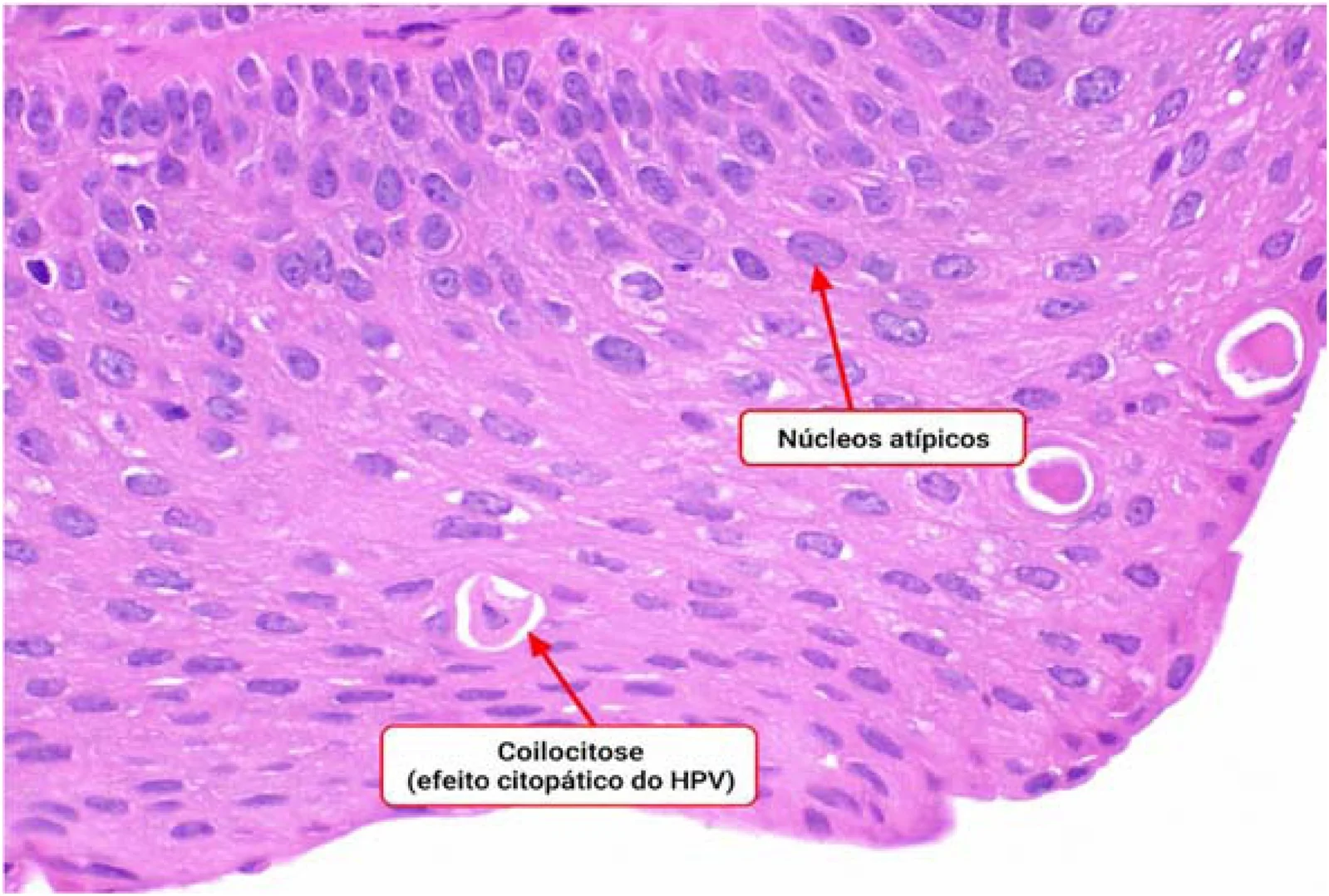
Histological image of the patient. Source: Personal collection.

### Tracheostomy

Four patients (23.53%) required tracheostomy due to the severity of their airway obstruction and the frequent recurrence of the disease. Among the patients who underwent tracheostomy, one presented extensive tracheal dissemination and required multiple interventions in a short period.

### Adjuvant therapies

A patient with extensive tracheal involvement received intralesional and systemic bevacizumab at another institution due to the clinical severity (image above).

After the adjuvant therapy, decannulation was possible, although new recurrences were observed subsequently; however, a new tracheostomy was not necessary, and quarterly bevacizumab injections were performed.

### Complications and mortality

Two patients died due to complications of the disease.

One of the cases occurred in a tracheostomized patient after accidental loss of the cannula and unsuccessful attempts to reestablish the airway in the emergency department.

Another patient developed severe respiratory failure prior to tracheostomy.

No cases of malignant transformation were identified during the follow-up period.

### Derkay classification

The severity of Recurrent Respiratory Papillomatosis (RRP) was quantified by the Derkay severity staging system,^8^ which is based on the anatomical location and progression of the lesions (anatomic assessment) and on their impact on the patient's general condition (clinical assessment). According to the Derkay system, the aerodigestive tract is divided into 25 sub-sites, each scored from 0 to 3 (0, Absent; 1, Superficial lesion; 2, Raised lesion; 3, Bulky lesion). The 25 scores are calculated to generate a composite anatomic score. The complementary clinical assessment takes into account the patient's voice (score 0–2), the occurrence of stridor (score 0–2), the urgency of the consultation (score 0–3) and the level of airway safety (score 0–4). The anatomic and clinical scores were then combined. In this study, the clinical assessment scores were obtained on the day before surgery. The Derkay clinical assessment was performed by the surgeon responsible for the operation and the Derkay anatomic score was calculated by the same person during the surgical procedure.

Base: 53 Derkay evaluations in 17 patients with complete data.

The Derkay score was assessed in 17 patients, totaling 53 endoscopic evaluations over the follow-up period (median of 3 evaluations per patient). The mean total score at the first evaluation was 10.94 points (12.7% of the maximum possible score of 86), decreasing to 7.00-points (8.1%) at the last available evaluation, which corresponds to a mean reduction of 5.58-points between the first and the last evaluation. Among the 12 patients with more than one recorded evaluation, a score reduction was observed in 11 (91.7%) and stability in 1 (8.3%), with no cases of worsening during follow-up. This progressive reduction in the score is consistent with the expected effect of repeated surgical treatment, which promotes successive excision of the lesions and the consequent decrease in tumor burden over the follow-up.

Regarding the anatomical distribution of the lesions, the mean number of sites affected per patient was 4.57 (18.3% of the 25 sites evaluated). The sites most frequently affected were the left true vocal fold and the anterior commissure, both present in 100% of patients over the follow-up, followed by the right true vocal fold (94.1%).

## Discussion

Juvenile Recurrent Respiratory Papillomatosis (RRP) remains a disease of high morbidity, particularly in young children, in whom airway involvement may rapidly progress to respiratory failure. In this retrospective series, we observed a high recurrence rate, frequent need for multiple surgical procedures and occurrence of severe complications, including tracheostomy and death.

The mean age at symptom onset observed in our study was similar to that described in the international literature, which reports a higher frequency of presentation before five years of age. Dysphonia was the predominant initial symptom, reinforcing its role as the most frequent clinical manifestation of the disease. A male predominance has been proposed in papillomatosis.^2^^,^^9^ In our study, however, we observed a predominance of the female sex.

However, a significant proportion of patients already presented with dyspnea at the time of specialist diagnosis, suggesting a delay in referral for otolaryngological or head and neck surgical evaluation. This finding may reflect initial diagnostic difficulties in young children, who are frequently initially treated for more common respiratory diseases. The fact that they have proportionally smaller dimensions of the pediatric larynx, particularly in the subglottic region, may be implicated as a cause of obstruction at less advanced stages of the disease, as well as the delay in diagnosis, difficulty in performing laryngoscopy and delay in referral to the reference center.^9^

The recurrence rate observed was high, consistent with the chronic nature of the disease described in other clinical series. The need for multiple surgeries represents a significant functional, emotional and socioeconomic impact for patients and their families. Dysphonia was present in 100%.

In our service, all resections were performed using conventional techniques with cold instruments, since a CO_2_ laser was not available during the study period. Although the laser is frequently described as the preferred technique in some centers, its unavailability remains a reality in many Brazilian public hospitals. It is assumed that the isolated presence of HPV is not sufficient to cause the disease. HPV has been identified on the vocal cords of patients without papillomatosis,^9^ suggesting that an additional event may be necessary to produce clinical HPV infection, such as malnutrition, immunosuppression, or concomitance of other viral infections.^10^ HPV appears to migrate from mucosa with normal appearance to areas of trauma (squamociliary junction) caused by prior excision, promoting recurrence.^11^ Steinberg et al. observed that the HPV genome can be found both in affected tissues and in adjacent tissues with normal appearance. Possibly, these normal areas serve as a source for reinfection, leading to recurrences.^10^, ^11^, ^12^ The prevalence of infection by this virus in the two forms of papillomatosis has not yet been clearly defined, nor has the healing rate after surgical trauma. Perhaps re-epithelialization by adjacent tissues is faster in younger individuals, which could contribute to the earlier recurrence observed in the juvenile group.^9^^,^^13^^,^^14^

The incidence of tracheostomy in patients with recurrent laryngeal papillomatosis reported in the literature ranges from 1.8% to 64%.^2^^,^^14^ In our series, approximately 24% of patients required tracheostomy before resection of the papillomas. This surgery may be associated with dissemination of the disease to the trachea, bronchi, and lungs.^15^^,^^16^ The need for tracheostomy occurred in a significant proportion of the patients evaluated, reflecting the severity of respiratory compromise in aggressive cases. The literature describes an association between tracheostomy and distal dissemination of the disease; however, these patients usually already present with clinically more severe forms, making it difficult to establish a direct causal relationship. Despite these differences, most authors agree that, when possible, tracheostomy should be avoided and, when necessary, decannulation should be scheduled as early as possible.

Regarding treatment, several methods have been described, including the use of antibiotics, hormones, electrocoagulation, cryotherapy, radiotherapy, ultrasound, surgical excision, autogenous vaccine, intralesional application of cidofovir, intralesional and parenteral administration of bevacizumab, and the use of a microdebrider for lesion removal.^7^^,^^9^^,^^14^^,^^17^^,^^18^ The use of bevacizumab as adjuvant therapy was observed in one patient with extensive tracheal involvement. Recent studies have shown promising results with antiangiogenic therapies in aggressive cases of recurrent respiratory papillomatosis, particularly in reducing the frequency of surgical interventions. However, access to such therapies remains limited in most public health systems. Two patients died in our series, emphasizing the potential severity of the disease, particularly when associated with significant respiratory obstruction. Although rare, RRP-related mortality has been described in the literature, mainly in patients with aggressive forms and distal dissemination.

Contrary to what may occur in papillomatosis in adults, no case of squamous cell carcinoma was identified in patients with the juvenile form and a prior diagnosis of recurrent laryngeal papillomatosis. However, it cannot be stated that malignant transformation in papillomatosis occurs exclusively in association with the presence of HPV, or whether it is a parallel phenomenon associated with other carcinogenic stimuli to which the larynx is exposed. Cofactors capable of modifying cellular genes and/or intracellular regulators, such as alcohol, tobacco, and other viruses, may act as initiators responsible for oncogenesis. The association between HPV and laryngeal squamous cell carcinoma remains under investigation.^19^

## Conclusion

Juvenile recurrent laryngeal papillomatosis is a disease that is frequently benign, with high morbidity and a tendency to recurrence and progression. Juvenile recurrent respiratory papillomatosis showed high morbidity in this case series, characterized by frequent recurrences, the need for multiple surgical interventions, and the occurrence of severe complications, including tracheostomy and death.

Disease management remains challenging in public tertiary care services, especially in locations with limited access to viral typing, specialized pediatric bronchoscopy, and adjuvant therapies. The use of the Derkay classification aids in staging the disease and may help predict the need for adjuvant therapy.

In more aggressive cases, tracheostomy was required to manage severe airway obstruction; however, tracheostomy-related complications contributed to mortality in this series, reinforcing that decannulation should be scheduled as soon as the patient's condition stabilizes, and the lesions remain resolved. In the juvenile form early recurrences associated with dyspnea occur, requiring repeated interventions. Cases of juvenile recurrent papillomatosis can be treated with adjuvant therapies, such as bevacizumab. The lack of specific tests in the Sistema Único de Saúde (SUS) for the detection of HPV subtypes limits the ability to identify patients with more aggressive subtypes and the inclusion of new subtypes in vaccines. Treatments that increase the interval between recurrences are needed, such as vaccinating children with recurrent laryngeal papillomatosis. Multicenter studies with larger samples are necessary to better understand prognostic factors and the potential impact of HPV vaccination on disease incidence.

During the preparation of this work, the author(s) used Claude (Anthropic), an artificial intelligence assistant, to assist with descriptive statistical analysis of the data, internal consistency checks of the text, and formatting review of the bibliographic references. The author(s) reviewed and edited the output as needed and take(s) full responsibility for the content of the published article.

## ORCID ID

Dário Oliveira Lopes: 0000-0002-7952-7328

Alana Mendonça Amorim: 0000-0002-5946-7700

Tiago Silva Almeida: 0009-0009-7215-2728

## Funding

This research did not receive any specific grant.

## Data availability

The authors declare that all data are available in repository.

## Declaration of competing interest

By their signature, all authors affirm that there is no actual or potential conflict of interest including any financial, personal or other relationships with other people or organizations within three years of beginning the submitted work that could inappropriately influence, or be perceived to influence, their work. If case such conflicts of interest exist, a disclosure document is signed by concerned authors.
